# Sensorimotor Integration in Childhood Dystonia and Dystonic Cerebral Palsy—A Developmental Perspective

**DOI:** 10.3389/fneur.2021.668081

**Published:** 2021-07-23

**Authors:** Verity M. McClelland, Jean-Pierre Lin

**Affiliations:** ^1^Department of Basic and Clinical Neuroscience, Institute of Psychiatry, Psychology and Neuroscience, King's College London, London, United Kingdom; ^2^Children's Neurosciences Department, Evelina London Children's Hospital, Guy's and St Thomas' NHS Foundation Trust, London, United Kingdom

**Keywords:** dystonia, children, dystonic cerebral palsy, sensorimotor integration, plasticity, critical windows, neurodevelopment, neuromodulation

## Abstract

Dystonia is a disorder of sensorimotor integration, involving dysfunction within the basal ganglia, cortex, cerebellum, or their inter-connections as part of the sensorimotor network. Some forms of dystonia are also characterized by maladaptive or exaggerated plasticity. Development of the neuronal processes underlying sensorimotor integration is incompletely understood but involves activity-dependent modeling and refining of sensorimotor circuits through processes that are already taking place *in utero* and which continue through infancy, childhood, and into adolescence. Several genetic dystonias have clinical onset in early childhood, but there is evidence that sensorimotor circuit development may already be disrupted prenatally in these conditions. Dystonic cerebral palsy (DCP) is a form of acquired dystonia with perinatal onset during a period of rapid neurodevelopment and activity-dependent refinement of sensorimotor networks. However, physiological studies of children with dystonia are sparse. This discussion paper addresses the role of neuroplasticity in the development of sensorimotor integration with particular focus on the relevance of these mechanisms for understanding childhood dystonia, DCP, and implications for therapy selection, including neuromodulation and timing of intervention.

## Introduction

**Figure 1:**
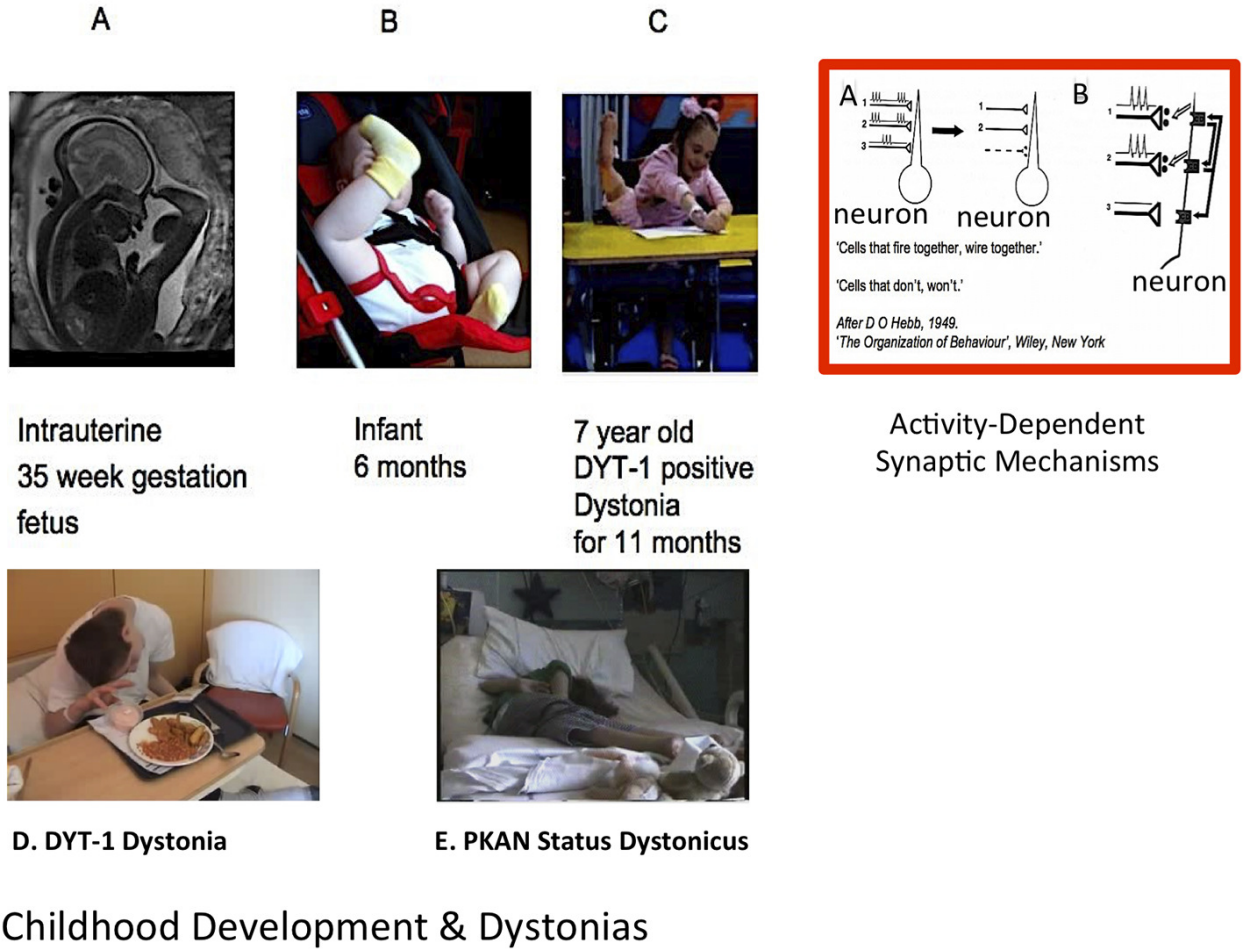
Normal development, dystonia, and role for activity-dependent plasticity. [**Top** (L-R)]: Thirty-five week gestation fetus; typically developing 6 month old infant; 7 year old girl with DYT-1 dystonia (2); Red Rectangle: **(A)** Example of activity-dependent synaptic plasticity. The two synchronously firing neurons (1 and 2) are retained by a “retrograde messenger” from the post-synaptic receptors shown in **(B)** while the asynchronously firing neuronal connection (3) is lost. From Penn and Shatz (3) annotated from *DO Hebb 1949: “The Organisation of Behaviour”*. [**Lower** (L-R)]: Adolescent with generalized DYT-1 Dystonia attempting to eat; Adolescent in status dystonicus due to Pantothenate Kinase associate Neurodegeneration (PKAN); *Clinical cases courtesy J-P Lin*.

Dystonia is a neurological syndrome characterized by involuntary, sustained, or intermittent, muscle contractions causing abnormal, often repetitive, movements, postures, or both. Dystonic movements are typically patterned, twisting, and associated with overflow muscle activation (1). Healthy babies and infants also express involuntary, sustained, patterned, and repetitive contraction of non-synergistic muscles, causing twisting movements or postures (Figure 1) which are replaced through development by skilled, purposeful, and economic movements (2). Although the underlying pathophysiological mechanisms of dystonia are still not fully understood, several common themes have emerged from physiological studies, including reduced inhibition within the CNS (4), exaggerated plasticity (5, 6), abnormal patterns of basal ganglia neuronal firing (7–10), and enhanced low-frequency oscillatory activity in the basal ganglia (11, 12), and an abnormal excessive low frequency drive to muscles (13–16). Another striking feature is aberrant sensorimotor processing, with the implication that distorted perception of incoming afferent information and its abnormal integration with motor commands leads to excessive and undesired dystonic movements (16–20). Abnormal sensorimotor processing, particularly measures of spatial or temporal tactile sensory discrimination, appears to be an “endophenotype” in certain genetic dystonias, being observed also in non-manifesting DYT1 carriers (21) or asymptomatic first degree relatives of patients with adult-onset primary torsion dystonia (22).

Unfortunately, despite the onset of many genetic dystonias in mid-childhood (e.g., DYT1 and DYT6), neurophysiological studies in children with dystonia are sparse (23). This leaves a significant gap in our knowledge of the impact of these genetic abnormalities on the sensorimotor system as it develops and matures through infancy, childhood, and adolescence, and during critical windows of neurodevelopment (23, 24) when neuroplasticity is naturally heightened compared with adults, facilitating development, learning, and adaptation in typically developing children (24). As will be seen below, these mutations may have an impact on sensorimotor development even *in utero*. Moreover, many acquired dystonias arise from brain injury in the perinatal period [i.e., dystonic cerebral palsy (DCP)], when the presence of transient neuronal structures or critical periods of synaptic plasticity and activity-dependent refinement of neuronal circuits gives rise to specific periods of vulnerability to insult (2, 24–26). To gain a thorough understanding of these processes, and the options for therapeutic intervention, it is important to study neurophysiological mechanisms of disease in infants and children rather than extrapolate from adult studies (23).

This article brings together literature on the neurophysiology of normal development of the sensorimotor system in the pre-natal and perinatal periods, childhood and adolescence, to consider the role of plasticity mechanisms during these times, and then to consider how these processes are relevant to dystonia, with an emphasis on both isolated genetic dystonias with onset in childhood and DCP.

## Neuroplasticity and Its Mechanisms

Neuroplasticity refers to the dynamic biological capacity of the central nervous system to undergo structural and functional change in response to experience, and to adapt following injury (24). These experience-driven changes are mediated by a variety of mechanisms, including neurogenesis, apoptosis, synaptogenesis, and synaptic pruning, as reviewed in detail elsewhere (24, 27). Two main forms of plasticity are described–Hebbian and homeostatic–and both play a critical role in nervous system maturation.

### Hebbian Plasticity

Hebbian mechanisms include changes in synaptic strength mediated via long-term potentiation (LTP) or long-term depression (LTD) following high frequency or prolonged, patterned pre-synaptic stimulation, respectively (28). See also Figure 1. LTP or LTD can also be induced by repeated pairing of single presynaptic stimuli with post-synaptic depolarisation, with the relative timing of the pre- and post-synaptic action potentials determining the direction of synaptic change (28). If Hebbian forms of plasticity are unregulated, there is the potential for neural circuit activity to become unstable and to “runaway” toward hyperactivity or quiescence (29). Homeostatic plasticity mechanisms are therefore required to guard against this possibility and stabilize the system.

### Homeostatic Plasticity

Homeostatic mechanisms regulate both synaptic numbers and synaptic strength (30) and act to adjust neuronal firing in response to changes in post-synaptic activity. Thus, when cortical networks are deprived of activity, network properties are altered to promote excitability, for example by an increase in strength of excitatory synapses onto excitatory neurons (29). In contrast, an elevation in network activity leads to a reduction in the strength of excitatory synapses. Homeostatic mechanisms thus restore activity to a “set-point” following perturbations (30) and act throughout the nervous system, both centrally and at the neuromuscular junction (29), thus tuning the central and peripheral mechanisms for action.

These homeostatic changes in synaptic strength occur relatively slowly over several hours and are mediated in various ways, such as proportional scaling of synaptic currents (i.e., each synapse is strengthened or weakened in proportion to its initial strength, allowing the relative differences between synapses to be preserved), changes in the clustering of postsynaptic receptors (i.e., prolonged synaptic inactivity leads to an increase in the insertion of post-synaptic receptors), presynaptic transmitter release or reuptake and changes to the number of functional synapses (29). Combinations of these mechanisms can also allow “sliding plasticity thresholds,” which adjust the ease with which LTP and LTD can be induced in an activity—dependent manner (29).

## Neuroplasticity and Dystonia

### Neurophysiological Tools Used to Assess Plasticity

The paired associative stimulation (PAS) protocol involves coupling of low frequency electrical peripheral nerve stimulation with transcranial magnetic stimulation (TMS) over primary motor cortex. This leads to an increase or decrease in the amplitude of the motor evoked potential (MEP), depending on the inter-stimulus interval between the peripheral and TMS stimuli, reflecting an increase or decrease in excitability of the corticospinal neurons (31). These effects are both enduring and show topographical specificity, and are therefore considered to represent LTP-like and LTD-like neuroplasticity within the sensorimotor system (31). Paired associative stimulation has been demonstrated in adults (31, 32) and in children from age seven upwards (33). The theta burst stimulation (TBS) paradigm applies bursts of high frequency TMS pulses to the motor cortex, each burst comprising three pulses at 50 Hz, with the bursts repeated at 5 Hz intervals. Intermittent TBS (comprising runs of 2 s on and 8 s off, repeated 20 times) gives rise to MEP facilitation (an LTP-like response), whereas continuous TBS for 40 s gives rise to an LTD-like response with suppression of MEP amplitude (34). The TBS paradigm therefore assesses plasticity in the motor cortex. Plasticity in the sensory cortex can be induced using high frequency repetitive somatosensory stimulation (HF-RSS), a protocol which applies patterned cutaneous electrical stimulation, and which can improve both two-point spatial discrimination and temporal discrimination in the stimulated area in healthy adults (35).

### Abnormalities of Hebbian and Homeostatic Plasticity in Adults With Genetic or Idiopathic Dystonias

Adults with focal hand dystonia show abnormal LTP-like plasticity, as measured with PAS, with a larger and less focal increase in corticospinal excitability compared with controls (5, 36) which could represent an over-recruitment and “blurring” of the distinct functions and characteristics between primary (SI) and secondary (SII) somatosensory cortices and primary and supplementary motor (MI and MII, respectively) cortices. This in turn can be related to dystonic symptoms, in which there is a loss of specificity in muscle activation, with overflow to other muscles, including antagonists. Additionally, excessive LTP-like plasticity may strengthen inappropriate sensory-motor associations, particularly with excessive practice or training, as is seen in musician's dystonias or writer's cramp (5). Abnormal plasticity is also present in adults with idiopathic dystonia or isolated DYT1 genetic dystonia, as demonstrated by enhanced long-lasting effects of TBS (37), while homeostatic plasticity, assessed with combined repetitive TMS and transcranial direct current stimulation, is abnormal in patients with focal hand dystonia (38). Furthermore, cerebellar modulation of motor cortex plasticity is impaired in patients with writer's cramp (39) and sensory cortex plasticity is abnormal in patients with cervical dystonia, in whom a paradoxical response to HF-RSS was seen, with a deterioration in somatosensory temporal discrimination (40).

**Figure 2:**
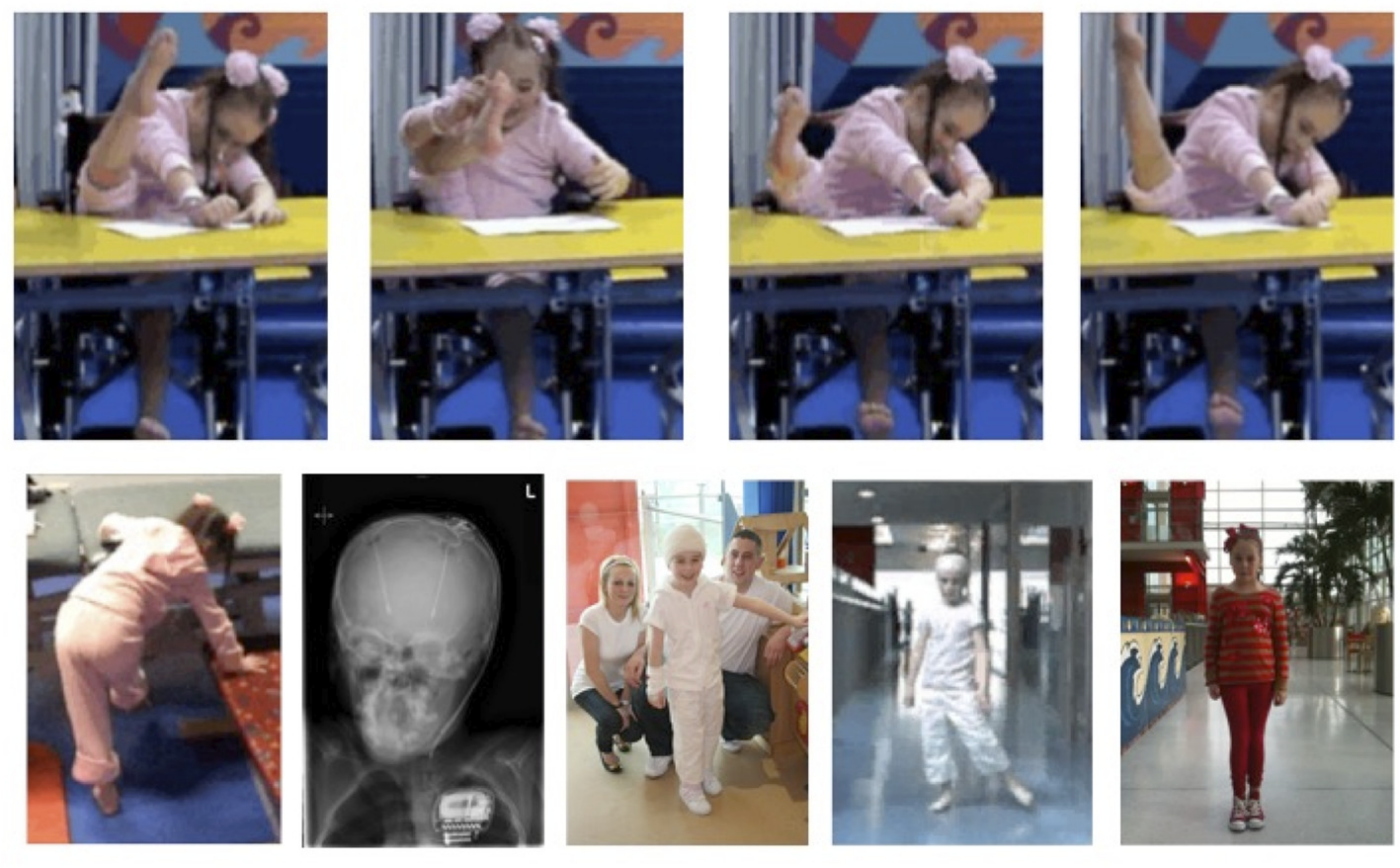
DYT-1 dystonia and intrusive “ballerina postures” and improvements with Deep Brain Stimulation (DBS). Video frames over time before and after DBS. Onset age 6.0 years with rapid progression to wheel-chair mobility by age 6.5 years. **(Top)** “Ballerina posturing” of the right leg interferes with lying and sitting (see also Figure 1C). [**Lower** (left-right)]: unable to stand or walk; head and chest X-ray post ACTIVA RC rechargeable DBS implant, age 7 years; standing unsupported within days of DBS; walking unsupported 3 months post DBS with a left circumducting gait; fully recovered at 3 years post-DBS. *Courtesy J-P Lin*.

Quartarone et al. postulated that abnormal or excessive cortical plasticity may lead to the consolidation of incorrect motor programmes (or “engrams”) containing redundant muscular activation, which in turn are manifest as overt dystonic movements (5). Examples are shown in Figure 2. Interestingly, restoration of plasticity levels (measured with PAS) toward those seen in controls is observed in patients who have responded clinically to pallidal Deep Brain Stimulation (DBS) (41, 42). The observation that improvement in dystonia occurs in proportion to the reduction in cortical plasticity implies that this effect on plasticity makes an important contribution toward the mechanisms of GPi DBS in dystonia (43), in turn suggesting an important pathophysiological role of the exaggerated plasticity response.

### Assessment of Plasticity in Acquired Dystonia

In contrast to idiopathic or genetic dystonias, patients with acquired hemidystonia have shown normal responses to both PAS (44) and HF-RSS (45), suggesting that abnormal plasticity, at least in the form measured with these particular protocols, may not be essential for the development of dystonia (45). On the other hand, in patients with acquired dystonia secondary to basal ganglia lesions, there is usually a delay between the time of injury and the onset of dystonic symptoms (46), which would be consistent with a maladaptive plasticity response.

How do we explain this conundrum? Examining the developmental origins of these disorders and how each may impact on sensorimotor circuit development may help to reconcile these findings. In this context it is also important to appreciate that, as well as plasticity generally being greater in early life, the site and extent to which various homeostatic plasticity mechanisms are active alters during different stages of neurodevelopment (30). For example, genetic dystonias, although often not manifesting until mid-childhood or later, are likely to influence development even during embryogenesis, when circuits are initially forming. In contrast, many acquired dystonias arise from injury in the perinatal period (DCP) when prior embryonic development of sensorimotor circuits has been normal, but is then impaired abruptly at a critical time when activity-dependent refinement of sensorimotor circuits would normally be heightened. Understanding the role of neuroplasticity mechanisms in the developing sensorimotor system may therefore provide critical insights in this regard.

## Prenatal and Perinatal Periods are Critical For Establishment and Experience-Driven Refinement of Sensorimotor Circuits

During embryonic and fetal development, thalamocortical neurones destined for sensory cortex pass via the transient structure of the sub-plate, and subsequently to the cortical plate, reaching the cortex around 24 weeks gestational age (25). Whilst the sub-plate is considered fundamental in establishing thalamo-cortical connections, more detailed connectivity depends on neuronal activity, including endogenous neural activity generated by the nervous system itself before sensory input is available (3).

### Evidence for the Role of Neuronal Activity in Sensorimotor Circuit Development

In humans, endogenously generated bursts of neuronal activity are seen in preterm infants from 26 weeks gestational age. From 28 weeks, “delta brushes” are seen, comprising slow delta waves (0.3–2 Hz) with superimposed fast (8–25 Hz) activity (47, 48), initially over central regions, but with the topography and predominant frequencies changing with gestational age (49). Delta brushes occur spontaneously, but can also be triggered by contralateral limb movements or somatosensory stimulation with a somatotopic distribution (47).

Animal models, particularly rodents in which early postnatal stages are comparable to late fetal stages of human development (50), support the concept that neuronal oscillatory activity in the developing somatosensory cortex plays a role in establishing early networks (51–54). Newborn rats demonstrate several patterns of spontaneous and sensory stimulus-induced oscillatory neuronal activity in somatosensory cortex, including 1–2 s runs of alpha frequency (8–12 Hz) activity, termed “spindle bursts” due to their typical appearance (52). *In vitro* and *in vivo* studies suggest that sub-plate neurones are involved in the generation or amplification of spindle bursts, which are considered to be the correlate of human delta brushes (25, 50). As with delta brushes, spindle bursts in newborn rat primary somatosensory (S1) cortex are triggered by muscle twitches with a somatotopic distribution. Inactivation of peripheral input using lidocaine leads to a significant reduction in the occurrence of spindle bursts in contralateral barrel cortex of newborn rats (54), indicating that a substantial proportion of spontaneous spindle burst activity in developing sensory neocortex is triggered by events in the sensory periphery (50, 54).

Recent fMRI studies show a somatotopic organization of sensorimotor cortex even in preterm human infants (55), suggesting early intrinsic determination of a broad somatotopic map. This is supported by the topography of sensory evoked responses to tactile stimulation of the upper and lower limbs in preterms (56). However, the fine-tuning of somatotopic organization within sensory and motor cortices requires experience-dependent synaptic plasticity. For example, in the rat, neonatal asphyxia and hind limb immobilization leads to disorganization of primary somatosensory (S1) cortex with abnormally large and overlapping hind limb receptive fields (57) indicative of a lack of activity-dependent synaptic pruning. Taken together the above observations suggest that early oscillatory neuronal activity, both spontaneous and sensory-evoked, plays an important role in the development and refinement of sensorimotor networks.

### Evidence of Activity-Dependent Refinement of Efferent Projections

During this same time period (24–34 weeks GA), efferent projection tracts are also developing, connecting the cortex with sub-cortical nuclei, cerebellum, and spinal cord (58). The corticospinal projections are initially bilateral but, during typical development, the ipsilateral projection is gradually withdrawn though a neuroplastic process of activity-dependent competition, particularly during the first 15–18 months after birth (59). The “rewiring” of descending motor pathways in individuals with hemiplegic CP provides another example of how activity, or lack of activity, during critical developmental windows shapes the development of structural and functional sensorimotor connections. Unilateral brain lesions disrupt the usual process of activity-dependent competition between ipsilateral and contralateral corticospinal projections, such that uncrossed ipsilateral projections from the non-lesioned hemisphere survive and are strengthened due to lack of competition from contralateral corticospinal fibers arising from the lesioned hemisphere, which end up being withdrawn (59, 60). The pattern of re-organization and relative strength of descending ipsilateral and contralateral projections varies depending on the timing and extent of the lesion (61, 62) and have been reviewed in detail elsewhere (58). However, the ascending somatosensory pathway retains its projection to the contralateral sensory cortex (63) and may even take a longer route to bypass a periventricular lesion and reach its intended destination in the postcentral gyrus (64).

It is clear that the prenatal and perinatal periods are critical for both the establishment and the experience-driven refinement of sensorimotor circuits. A pathological process that disrupts the normal pattern of events during these periods will therefore have a profound effect on sensorimotor development, whether it be via genetically determined abnormalities of synaptogenesis and synaptic plasticity, or due to a lack of the normal activity responsible for experience-dependent synaptic modification and the consequential effect on homeostatic plasticity set-points.

## How are Synaptic Plasticity and Experience-Driven Refinement of Sensorimotor Circuits in the Prenatal and Perinatal Periods Relevant to Genetic Dystonia?

There is growing evidence that several genetic dystonias are associated with abnormal synaptogenesis and synaptic plasticity.

### DYT1 Dystonia

One well-studied example from the myriad of genetic dystonias is DYT1, a severe generalized dystonia with typical onset in mid-childhood (Figures 1C,D, 2), resulting from a mutation in *TOR1A*, (typically a three base pair deletion leading to loss of a glutamic acid residue at the carboxyl-terminal region). This mutation leads to impaired function of the protein TorsinA which is thought to pay a role in the function of the endoplasmic reticulum and nuclear envelope as well as in interactions between the cytoskeleton and nuclear membrane and regulation of cellular lipid metabolism (65). These are all key processes in synaptogenesis and synaptic plasticity, since changes in the numbers of post-synaptic receptors involves changes in the turnover and synaptic localization of many post-synaptic scaffolding proteins (29). Mouse models of TorsinA hypofunction display altered protein homeostasis and abnormal synaptic transmission and plasticity (66, 67). One model has shown subtle neurodegeneration in several regions of the sensorimotor network, including sensorimotor cortex, thalamus, globus pallidus, and deep cerebellar nuclei (67). It is noted that the double knock-out model used in the Liang study is genetically quite different from DYT1 patients, who usually have a single heterozygous mutation, and that other studies using *Dyt1* heterozygous knock-out mice have not demonstrated neurodegeneration (66). However, evidence from several animal model studies indicates that TorsinA hypofunction affects multiple regions of the sensorimotor network and that there are early critical windows in which sensorimotor circuit development may be vulnerable to TorsinA hypofunction, particularly during periods of intense synaptogenesis (67–70).

### DYT6 Dystonia

Mutations in *THAP1*, a zinc-finger transcription factor, which lead to DYT6 dystonia, have also been associated with dysfunction of molecular pathways leading to defective neuritogenesis, with implications for axonal guidance, and defects in synaptic plasticity (71). Although these deficits were observed in early development of the mutant mouse, Zakirova et al. raise the interesting suggestion that any effects on neuritogenesis that persist into adulthood might be offset by the synaptic changes (71). Interestingly these abnormalities show convergence with those disrupted in DYT1 dystonia i.e., in both DTY1 and DYT6 there is a defect of neuritogenesis and synaptic plasticity (71). In human functional imaging studies, both DYT1 and DYT6 are associated with abnormal connectivity in cerebello-thalamo-cortical pathways (72). Importantly, these findings emphasize that the disrupted plasticity and activity in the sensorimotor network caused by *TOR1A* or *THAP1* dysfunction are not just manifest at the time of clinical onset (usually mid-childhood) but are likely to impact on the early development of sensorimotor circuits and also on their function, perhaps shifting the balance between excitation and inhibition.

### Relevance of Abnormal Synaptic Plasticity in Genetic Dystonias to Sensorimotor Network Development

Neuroplasticity is generally enhanced during development compared with adulthood (24). In addition, the extent to which various plasticity mechanisms are operative fluctuates during development and across different parts of the brain (30). In humans, synaptogenesis starts around 27 weeks gestational age and intensifies during the first 2 years of life, with maturation patterns varying across different cortical regions (24). Abnormal synaptic plasticity within the sensorimotor network due to *TOR1A* hypofunction is therefore likely to have a significant impact during early sensorimotor development, including prenatally. Synaptic pruning also peaks at different times for different brain regions, earlier in auditory than in pre-frontal cortex, the latter continuing into adolescence (24). Furthermore, the pattern of expression of CNS receptors changes with a sequential (but partly overlapping) expression of GABA-A, then NMDA and then AMPA receptors, supporting the development of LTP and LTD and a transition from neonatal to adult forms of plasticity (73–75).

Although originally identified in DYT1 dystonia, there is now evidence of an association between variant mutations within the *TOR1A* gene and focal dystonia and writer's cramp (65), disorders which tend to occur with overuse, and to present in adulthood. Further studies will be required to delineate the possible effect of these particular mutations on sensorimotor circuits during development (including prenatally) and the intriguing possibility of preventing manifestation of the disorder by modulating neuroplasticity in early life.

## How are Synaptic Plasticity and Experience-Driven Refinement of Sensorimotor Circuits in The Prenatal and Perinatal Periods Relevant to Dystonic Cerebral Palsy?

Dystonic cerebral palsy (DCP) refers to dystonia that arises from a brain insult in the perinatal period. This encompasses individuals with acquired dystonia due to hypoxic ischaemic-encephalopathy (HIE) at term (i.e., birth asphyxia), extreme prematurity, or unconjugated hyperbilirubinaemia-induced kernicterus, or indeed a combination of these.

### Patterns of Brain Injury in DCP

The patterns of brain lesions seen on MRI in DCP differ with timing of injury: typical MRI findings in HIE due to acute severe asphyxia reflect those areas of high metabolic rate at the time of injury, with classical involvement of the thalamus, basal ganglia (especially posterior putamen), peri-rolandic (sensorimotor) cortex, and sometimes also the cerebellum (76–79). The overlap between these areas and those implicated in the functional neuroanatomy of dystonia is striking (80–82). In extreme prematurity one is more likely to observe periventricular leukomalacia or the sequelae of intraventricular hemorrhage, but a normal structural MRI is reported in up to 50% of prematurely born children with DCP (83). In kernicterus, the injury on MRI is localized to the globus pallidus internus and subthalamic nuclei but is notoriously transient in many cases. Although imaging cohorts find “normal” structural MRI scan reports in 17–20% of children with CP (76–78), it is important to remember that changes to the *development and function* of sensorimotor networks are still likely to be present.

### Impact of Brain Injury on Sensorimotor Circuit Development in DCP

Although a broad somatotopic map is usually present in sensory cortex by term (or before), its further development is refined by experience-dependent connectivity changes.

#### Neonatal Asphyxia

For those with HIE, the development of sensorimotor circuits will have proceeded normally up until the time of injury, but then the injury to the thalamus, a crucial relay in the passage of sensory information to the cortex, and the subsequent disruption of thalamocortical pathway activity, will disrupt the on-going refinement of sensorimotor circuitry that usually occurs in the post-natal period. There is also injury to the sensorimotor cortex itself (peri-rolandic cortex) and to the basal ganglia and cerebellum, all important sub-cortical structures in the sensorimotor network. Thus, several nodes of the network may be disrupted.

As noted above, rat models demonstrate that neonatal asphyxia with hind limb immobilization leads to impaired somatotopic organization of primary sensory cortex with abnormally large and overlapping hind limb receptive fields (57) suggesting a lack of activity-dependent synaptic pruning. It is notable that in adults with focal or generalized dystonias, widened and overlapping sensory receptive fields have also been demonstrated, both in the cortex [e.g., (84) and in the globus pallidus (7)]. Even in the absence of asphyxia, early restriction of sensorimotor activity in newborn rats gave rise to maladaptive plasticity in the SM cortex (85, 86) with degradation-“blurring”- of the somatotopic organization of somatosensory cortex. Importantly, neuronal responses in sensorimotor cortex remained abnormal even after sensorimotor activity was no longer restricted (86), emphasizing the long-lasting impact of an insult within this critical window. Whilst equivalent experimental paradigms are not feasible in human infants, there is clear evidence from clinical studies that abnormal sensory evoked potentials (SEPs) have high predictive value for neurological sequelae in post-asphyxiated neonates (25).

#### Extreme Prematurity

In individuals with cerebral palsy born pre-term with periventricular white matter injury on MRI, diffusion tensor imaging studies show injury to the posterior thalamic radiation, indicating disruption of thalamocortical connections (87, 88). Moreover, the injury in the posterior thalamic radiation pathways is more severe than that in the descending corticospinal tracts and correlates with clinical measures of severity (88). These seminal findings emphasized the importance of disruption to sensory and not just motor pathways in cerebral palsy due to prematurity. As indicated by the animal studies outlined above, reduction or loss of peripheral input leads to reduced oscillatory activity in the developing sensorimotor cortex (52, 54). It can be envisaged that injury or dysfunction of the ascending sensory tracts of the posterior thalamic radiation will disrupt the passage of sensory information to the sensory cortex, and that the subsequent disruption of sensorimotor cortex neuronal oscillatory activity will in turn affect the development of sensorimotor circuits, even if the dysfunction of ascending pathways is only transient. Indeed, in preterm infants with structural brain lesions (bilateral intraventricular hemorrhage) somatosensory evoked responses to tactile stimuli are abnormal (56). Interestingly, the responses only became abnormal after a delay of several weeks (56), suggesting that the abnormality may reflect a neuroplastic response with re-organization of the relevant neuronal networks.

#### Role of Somatosensory Evoked Potentials

Importantly, abnormal somatosensory evoked potentials have a high predictive value for adverse neurodevelopmental outcome in both preterm infants and term infants with HIE (25, 89–92), with SEPs evoked from posterior tibial nerve stimulation having particular sensitivity for predicting development of CP in preterms (90, 91). In addition to the clear clinical application of these studies, the findings are also concordant with the notion that sensory pathway dysfunction has a negative impact on the normal activity-dependent development of sensorimotor networks.

In a study of upper and lower limb SEPs in young people with dystonia, 47% of patients showed an abnormality in at least one of their SEP responses (93). A similar proportion of 40% was confirmed in a larger cohort (94). The abnormalities were seen predominantly in the acquired dystonia group, of which the majority had dystonic-dyskinetic CP (93, 94). Although this was not a longitudinal study, it is likely that these abnormalities were long-standing and therefore that sensory pathway dysfunction had been present since the perinatal period in these individuals, with a consequent adverse impact on the activity-dependent refinement of sensorimotor circuits in the early post-natal period, and an enduring effect on their function (86).

## Sensorimotor Development in Childhood and Adolescence

Neuronal sensorimotor development continues throughout childhood and into adolescence and is associated with age-related improvements in sensorimotor skills, leading to a reduction in associated postures and unwanted overflow (95–98). The neurophysiological mechanisms of motor performance enhancement and refinement also reflect activity-dependent plasticity.

### Postnatal Cortical Oscillatory Activities and Reactivity

In humans, the post-natal development of cortical oscillatory activities and their reactivity to sensory stimulation is well-documented (99). A 6 Hz rhythm is seen over central regions from as early as 5–6 months (99). The peak frequency of this central rhythm increases with age, especially in the first year of life (99–101) when motor skills are developing rapidly. By the end of the first year of life, a 6–9 Hz rhythm is present over sensorimotor cortex which displays the typical characteristics of the adult mu rhythm (adult mu having a frequency of 8–12 Hz and a distinctive arciform morphology), showing suppression in response to movement and/or somatosensory stimulation (101, 102). Thus, mu event-related desynchronisation (ERD), which is considered to reflect sensorimotor processing (103–105), is present in infancy, at a time of rapid sensorimotor development. Stroganova et al. (100) observed that the peak frequency of mu rhythm in the second half-year of life, depended on the duration of intra- and extra-uterine development, whereas the frequency of the occipital alpha rhythm depended only on extra-uterine development i.e., the presence of visual input, suggesting that the development of the sensorimotor mu rhythm itself is influenced by somatosensory stimulation, which is present even in the uterus (100).

### Mu Rhythm Modulation

The mu rhythm and its modulation continues to develop throughout childhood, with the mu/alpha ERD being followed by a mu/alpha *event related synchronization* (ERS) (20) and the emergence of a beta range (14–30 Hz) ERD and ERS also in response to movement (20, 102, 106, 107). Whilst ERD is considered to reflect activation of sensorimotor networks, ERS is considered to reflect active inhibition or “resetting” within the sensorimotor cortex (103–105). Evidence from adult studies suggests that post-movement beta ERS may relate to erasing working memory information after task completion, or an integration between feedback and reward (108). Development proceeds further during adolescence before reaching an adult pattern (109). For example, recent observations suggest a relative “overshoot” in the mu/alpha ERS response in 10–14 year olds, which becomes more refined in 15–21 year olds (20). Similarly, in a recent MEG study of alpha-beta cortical oscillations in response to lower limb somatosensory stimulation applied during isometric force production, adolescents showed a more enhanced modulation compared with adults (109). Thus, the balance between excitation and inhibition may still be maturing at this stage.

### Patterns of Neuronal Oscillatory Activities in Dystonia

Dystonia is associated with enhanced low frequency (4–12 Hz) neuronal oscillatory activity in the basal ganglia (11, 12, 110) and also in the motor cortex (111). The exaggerated low frequency basal ganglia oscillations are coherent with activity in other parts of the sensorimotor network, including the cerebellum (12), correlate with symptom severity (112), and are coherent with dystonic EMG (14). An abnormal excessive low frequency drive to muscles has been shown in several forms of dystonia (13–16). The enhanced low frequency oscillations in the basal ganglia and motor cortex are suppressed by DBS (11, 111), as is the exaggerated low frequency muscular drive (15). Interestingly the performance of an effective sensory trick in two patients with cervical dystonia has been associated with a bilateral desynchronisation in the 6–8 Hz and beta band in both the globus pallidus and sensorimotor cortices (113), while a similar maneuver in two patients wtihout an effective sensory trick induced a worsening of dystonia and an increase in 6–8 Hz oscillations. These observations support the notion that exaggerated low frequency oscillations play an important role in the pathophysiology of dystonia.

**Figure 3:**
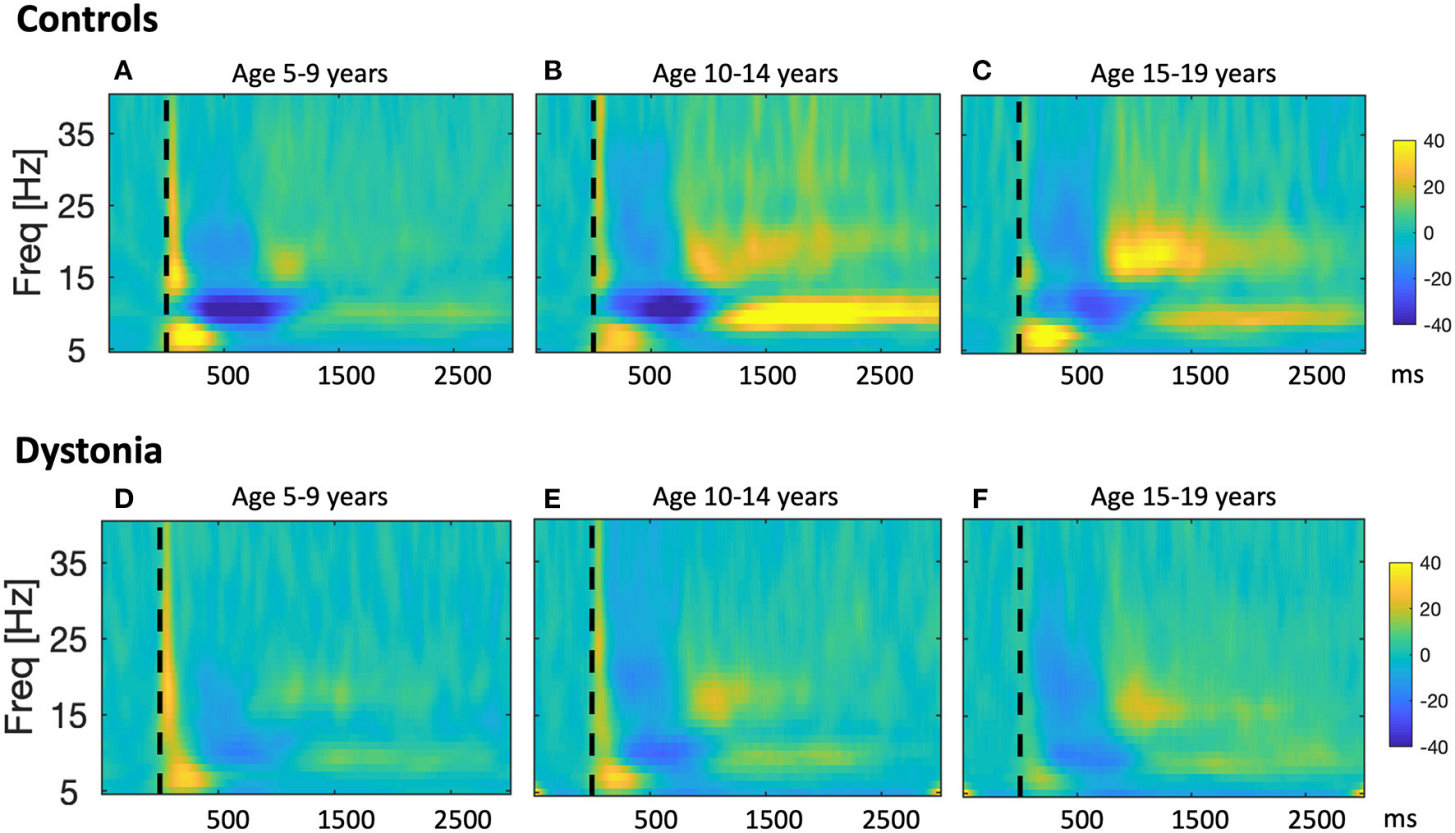
Developmental sequence of event-related changes in EEG power in relation to a proprioceptive stimulus in typically developing children and children with dystonia. Results from a study in which changes in sensorimotor cortex EEG were recorded in response to proprioceptive stimuli in 30 young people with dystonia and 22 controls (20). Participants sat at a table with their arm positioned in the arm-rest of a robotic wrist interface which delivered controlled passive wrist extension movements, resulting in brief stretches of the wrist flexors, with rise-time of 240 ms, and a target of 12° from the neutral position. Up to 160 wrist extension movements were recorded for each hand. Scalp EEG was recorded using a BrainVision system (BrainAmp MR Plus) and stimulus timing was synchronized with the EEG recordings via an electrical marker designating the movement onset. Offline, data were segmented into epochs comprising 1 s pre-stimulus and 3.5 s post-stimulus. After artifact rejection, remaining epochs were averaged to produce a stretch evoked potential for each hand in each subject. EEG power was calculated in 1 Hz bins from 5 to 40 Hz using the continuous Morlet wavelet transform with eight wavelet cycles. Relative changes in post-stimulus EEG power with respect to the pre-stimulus period were calculated. The figure shows pooled time-frequency plots across subjects showing the response over the contralateral hemisphere to stretch of the dominant hand wrist flexors i.e., right sensorimotor cortex for left hand movement, left sensorimotor cortex for right hand movement for controls **(A–C)**, and individuals with dystonia **(D–F)**, grouped by age. Left column: Young age group (5–9 years, *n* = 10), middle column: Intermediate age group (10–14 years, *n* = 6), right column: Older age group (15–19 years, *n* = 6). x-axis shows time in ms after the stimulus (dashed vertical line), y-axis shows frequency, color scale shows relative power at each frequency with respect to the pre-stimulus period, such that dark blue indicates event-related desynchronisation (ERD) and yellow-orange indicates event-related synchronization (ERS). The sharp increase in power with respect to baseline at time zero, extending up to 40 Hz, and the brief, early increase in theta range (4–7 Hz) power from 0 to 300 ms are likely to reflect movement artifact and a contribution from the stretch evoked potential, respectively. Figure adapted from McClelland et al. (20).

Much of this work involves invasive recordings and has therefore focused on adults with dystonia. The development of cortical and basal ganglia oscillatory activity in young patients with dystonia remains largely unexplored. Normal patterns of development of cortical oscillatory activities are well-documented, as noted above, with the proportion of low frequencies [delta (1–3 Hz) and theta (3–7 Hz)] generally decreasing with age, and the proportion of higher frequencies [alpha (8–12 Hz) and beta (14–30 Hz) ranges] generally increasing with age. A recent study found that the spectral content of scalp EEG over sensorimotor cortex in children with dystonia follows a similar general pattern with age, but that power in the theta range is relatively higher than in typically developing children (20). This study found that children with either isolated genetic dystonia or with DCP show impaired mu modulation in response to a proprioceptive stimulus, indicating an abnormality in sensorimotor processing which is common across different dystonia etiologies (20)—Figure 3. Importantly, this abnormality was present even in the youngest age-group tested (5–9 years). It is possible that this form of sensorimotor processing does not develop adequately in dystonia, either due to genetic abnormalities of synaptogenesis and synaptic plasticity (for example as in DYT1 or DYT6 dystonia) or due to lack of sensory input during a critical window of sensorimotor circuit development (e.g., in DCP). However, it is also possible that the observations reflect a secondary plastic change in the sensorimotor cortex due to abnormal feedback from dystonic muscle activity. This remains to be tested.

### Neurophysiological Phenomena Show Different Maturational Profiles

Different neurophysiological phenomena relevant to sensorimotor function show different maturational profiles. For example, TMS studies have shown that short-latency intracortical inhibition is present but at significantly lower levels in children under the age of 10 compared with adults (114). Intercortical inhibition, as measured by the ipsilateral silent period, is absent in pre-school children: it can be seen by age 6–7 years, but with a delayed latency and short duration compared with the adult ipsilateral silent period, and tends to mature by early adolescence (115, 116). Somatosensory gating, investigated using a paired-pulse electrical stimulus paradigm in children aged 10–18 years, appears to show a fully mature pattern by age 10 years (117). Interestingly it has been suggested that alpha/mu ERD may in part reflect somatosensory gating (118). The earlier development of mu ERD compared with mu or beta ERS (20) in this context is thus concordant with the suggestion that somatosensory gating mechanisms develop earlier than the (motor cortical) inhibitory mechanisms measured with TMS, or those reflected by movement related beta and gamma oscillations, which continue to develop during childhood and adolescence and into young adulthood (119). The lower levels of intracortical inhibition in children have been suggested to facilitate greater plasticity and motor learning during normal development (114, 120). In dystonia/DCP, these lower levels of inhibition may contribute toward a destabilization of pathologically enhanced plasticity at a critical stage in mid-childhood, but could also provide a window of opportunity for intervention.

## Relevance of Neuroplasticity and Sensorimotor Development for Timing of Neuromodulation Therapies

Overall, these different developmental disorders, all with the clinical manifestation of dystonia, are each characterized by disruption to the early development of sensorimotor networks, although at different stages and with different impacts on and responses from neuroplastic mechanisms.

### Timing of Injury and Disruption to Sensorimotor Development

Individuals with dystonic CP due to prematurity may experience a disruption of thalamocortical pathway activity, through damage to posterior thalamic radiation fibers, at a developmental stage when the subplate is still guiding the development of sensory cortex and somatotopic maps are still being formed. Those with dystonic CP due to term HIE will have had normal prenatal development of sensorimotor circuits followed by sudden and profound disruption of thalamocortical pathway activity and thus of the neuronal activity which would normally determine the on-going refinement of sensorimotor circuitry that occurs in the post-natal period. In addition, other nodes of the sensorimotor network will have been damaged (basal ganglia, cerebellum, sensorimotor cortex itself). Individuals with genetic dystonias (including for example *TOR1A* or *THAP1* related dystonias), although not manifesting clinically until mid-childhood, may actually have had an even earlier prenatal onset of sensorimotor circuit dysfunction, perhaps so early that functional circuits could still develop through compensatory mechanisms, as alluded to by Zakirova et al., with defects in neuritogenesis being offset by synaptic changes (71). One could speculate that an abnormality of dendrite formation leads to reduced neural activity at a critical time-point in prenatal development, which in turn leads to a change in the plasticity threshold due to homeostatic mechanisms, or even in the *range* of the plasticity response, which enhances the effect of LTP-like mechanisms. Whilst this enhanced LTP-like plasticity may initially be an advantage, there may come a point in mid-childhood, when LTP is enhanced as part of normal maturation, where the system is tipped out of a functional range and into an unstable range causing hyper-excitability i.e., the destabilizing effect of Hebbian plasticity is unchecked and leads to a driving of synaptic strengths toward maximum values.

In contrast, in acquired dystonia due to perinatal brain injury, the “plasticity machinery” itself may be intact (44, 45), but the consequence of reduced and atypical patterns of afferent feedback in early life induces a different form of maladaptive neuroplasticity. The “set-point” around which these processes function may well have been affected by the relative deprivation of afferent input induced by the lesion (30), even if this was only temporary. This can be considered a type of reactive plasticity, following sensory deprivation or CNS insult (24). Interestingly, a cTBS (continuous Theta Burst Stimulation) study demonstrated that adolescents who had been born pre-term showed a reduced LTD-like response to cTBS compared with adolescents born at term, suggesting that LTD-like neuroplasticity regulation is impaired in this group (121). These were not individuals with dystonia, but the study emphasizes that pre-term birth can have an impact on the function of plasticity mechanisms.

Thus, the ultimate effects of sensory deprivation on cortical circuitry are the result of a complex interplay between Hebbian and homeostatic forms of synaptic plasticity (29). These considerations could help to explain the conundrum presented earlier. For example, it is possible that patients with acquired dystonia may show normal PAS or HF-RSS responses, but that the set-point around which the underlying plasticity mechanisms are working has been changed due to the relative deprivation of sensory input resulting from the CNS insult.

### Timing of Intervention

The observation that there is often a delay between the time of insult and the development of symptoms in acquired dystonia suggests a maladaptive plasticity response and raises the exciting possibility that intervention within this time could be beneficial in preventing the development of dystonia. Even after the onset of dystonia, timing of intervention is important. Early presentation of dystonia in childhood is often followed by gradual worsening without remission despite attempted pharmacological and physical support interventions (122). There is evidence from clinical studies that the duration of dystonic symptoms or the proportion of life lived with dystonia has an influence on DBS response: outcomes from DBS showed a negative correlation with dystonia duration, normalized for age at surgery (123) and this relationship was observed for both primary (isolated genetic or idiopathic) and acquired dystonia (123). Marks and colleagues emphasize that DYT1 dystonia progresses quite rapidly during its early course, and that the initial goal of DBS is to arrest or slow this deterioration before actual improvement can occur (124), another factor in favor of early intervention.

A meta-analysis of 321 patients (from 72 articles) confirms that shorter duration of life lived with dystonia (or older age at onset) is associated with better improvement scores, as measured with the Burke Fahn Marsden Dystonia Rating Scale (125).

The foregoing review of time-critical events in the developing brain is sharply illustrated by the success of cochlear implantation to augment hearing (a special sensory input) in late infancy and early childhood with a view to promote language recognition and speech production, which has a greater chance of success if cochlear neuromodulation is performed before the age of 5 years when auditory and language plasticity windows are open (126). Comparable studies of intervention in early life for children with acquired dystonia (in particular DCP) are not yet available, with most reported cases of DBS surgery taking place from 5 years of age onwards (127). However, the findings above, regarding the relationship between proportion of life lived with dystonia and DBS response, combined with the documented success of early cochlear implantation, indicate that neuromodulation for movement disorders in childhood constitutes a race against time before the dysfunctional movement patterns and sensory experiences are neuroanatomically and neurophysiologically set (126).

Abnormal neuronal activity during critical windows in the prenatal and perinatal periods clearly has enduring effects on neural circuit activity for later life. This is demonstrated by the studies in rats, discussed above (85, 86), and also by studies in drosophila, in which increased neuronal excitation during a critical embryonic period could permanently induce seizure behavior in post-embryonic stages (128). An opportunity for “rescue” might be possible, but only during these critical early time-periods (128). Interestingly, in a study of neuromotor outcome in very preterm infants, Pike and Marlow describe a phenomenon of “transient dystonia” (or dystonia of prematurity), in which infants showed abnormalities of tone and dynamic function over the first postnatal year, but were neurologically normal by the age of 2 years. The authors speculated that these infants had sustained a less severe brain injury, but the observations could also suggest that these early years represent a period in which “rescue” is still possible through early intervention.

Very early intervention with neuromodulation, including DBS, in younger children will require not only the further development of surgical methods and age-appropriate equipment, but also, and indeed more importantly, a greater in-depth understanding of the pathophysiology of sensorimotor circuit development in infants with, or at risk of developing, dystonia/dystonic CP. Herein, lies a huge and critical gap in scientific knowledge which needs urgently to be addressed. Technology is advancing rapidly, but without this fundamental scientific understanding, trials of DBS neuromodulation in very young children and infants will not be possible or ethically acceptable. Other potential considerations include the use of non-invasive neuromodulation techniques to harness neuroplasticity within critical windows for sensorimotor circuit development. This could include non-invasive neurostimulation methods such as TMS, or targeted occupational or physical therapy techniques aiming to modulate neural activity by enhancing sensorimotor afferent input in infants who would otherwise experience a restriction or deprivation or sensory stimulation following a CNS insult. Such approaches could be used independently or as an adjunct to DBS, or could help to achieve a situation where the child is more likely to respond to DBS at a later stage, when the risk/benefit ratio of neurosurgery may be more favorable. Again, these potential interventions need to be based upon a robust understanding of the fundamental mechanisms and the critical time windows for neuroplasticity in sensorimotor circuit development in human infants. These studies need to be performed in order to establish a strong evidence-base and to provide the confidence and impetus for clinical trials of such early interventions.

## Conclusion

It is clear that critical windows exist, during which abnormal neural activity has enduring effects on future neuronal function and also during which restoration of normal activity might prevent long-term, persistent disruptions of neuronal circuit function (128). This is highly relevant to our understanding of dystonic CP and genetic dystonias with onset in childhood, but also provides insights into the mechanisms underlying dystonias with onset in later life. We are currently missing out on opportunities to intervene early and improve neuromotor developmental outcomes due to critical gaps in scientific knowledge and a sparsity of research in pediatric dystonia/dystonic CP. Further study is urgently needed to understand in detail how neuroplasticity processes influence sensorimotor circuit development and function in dystonic CP and to define the potential time windows in which intervening to modulate neural activity may induce a more normal pattern of development, either through invasive or non-invasive methods of therapy, or their combination.

## Author Contributions

VM wrote the first draft of the manuscript. J-PL wrote sections of the manuscript. All authors provided material for figures and read and approved the final version.

## Conflict of Interest

J-PL received unrestricted educational support for instructional courses and consultancy fees from Medtronic Ltd. The remaining author declares that the research was conducted in the absence of any commercial or financial relationships that could be construed as a potential conflict of interest.

## Publisher's Note

All claims expressed in this article are solely those of the authors and do not necessarily represent those of their affiliated organizations, or those of the publisher, the editors and the reviewers. Any product that may be evaluated in this article, or claim that may be made by its manufacturer, is not guaranteed or endorsed by the publisher.
